# Green Extraction of Orange Peel Waste Reduces TNFα-Induced Vascular Inflammation and Endothelial Dysfunction

**DOI:** 10.3390/antiox11091768

**Published:** 2022-09-07

**Authors:** Chen Huei Leo, Su Yi Foo, Joseph Choon Wee Tan, U-Xuan Tan, Chee Kai Chua, Eng Shi Ong

**Affiliations:** 1 Science, Math & Technology, Singapore University of Technology & Design, Singapore 487372, Singapore; 2 Pillar of Engineering Product Development, Singapore University of Technology & Design, Singapore 487372, Singapore

**Keywords:** orange peel waste, PHWE, LC/MS, anti-inflammatory, antioxidant

## Abstract

Orange peel waste (OPW) is known to contain an abundant amount of polyphenols compounds such as flavonoids, well-reported for their antioxidant and anti-inflammatory properties. While OPW is generally regarded as a food waste, the opportunity to extract bioactive compounds from these “wastes” arises due to their abundance, allowing the investigation of their potential effects on endothelial cells. Hence, this study aims to use a green extraction method and pressurized hot water extraction (PHWE) to extract bioactive compounds from OPW. Liquid chromatography with UV detection (LC/UV) and liquid chromatography mass spectrometry (LC/MS) were subsequently used to identify the bioactive compounds present. Through the optimization of the extraction temperature for PHWE, our results demonstrated that extraction temperatures of 60 °C and 80 °C yield distinct bioactive compounds and resulted in better antioxidant capacity compared to other extraction temperatures or organic solvent extraction. Despite having similar antioxidant capacity, their effects on endothelial cells were distinct. Specifically, treatment of endothelial cells with 60 °C OPW extracts inhibited TNFα-induced vascular inflammation and endothelial dysfunction in vitro, suggesting that OPW possess vasoprotective effects likely mediated by anti-inflammatory effects.

## 1. Introduction

Citrus fruits are one of the most consumed and cultivated fruits globally, with increasing projection of production to meet global needs in the future [1]. The increasing demand and consumption of citrus fruits generate citrus fruit wastes, which are disposed by incineration or utilised as animal feed. Citrus peels are composed of a coloured outer layer called flavedo, while the internal white, spongy layer is known as albedo [1]. While citrus peels are generally under-utilised, they are a rich source of bioactive compounds such as flavonoids and other polyphenolic compounds [2,3,4]. For example, the Washington navel oranges have been reported to contain relatively high concentration of the flavanones, hesperidin, and narirutin when compared to other citrus fruits [5,6]. Similarly, high concentrations of phenolic acids, such as ferulic and p-coumaric acids, were also reported in Valencia orange peels [5,6].

One of the biggest groups of naturally occurring polyphenolic substances, flavonoids, are well-recognised to possess several biological activities, including antioxidant, anti-inflammatory, and vasodilatory properties [7,8,9]. Given that oxidative stress and inflammation are common features reported in endothelial dysfunction, a critical and initiating factor for several vascular complications [10,11,12,13], flavonoids are widely considered as potential sources of natural product to exert beneficial effects on the cardiovascular system [14]. Indeed, several previous studies have shown that flavonoids present in orange peel waste (OPW) possessed antioxidant and anti-inflammatory properties [15,16] although less is known on the effects of OPW in endothelial cells. As the OPW represents a considerable proportion of the fruit, abundant in flavonoid content and yet often unconsumed and discarded, it creates an opportunity to explore suitable extraction methods to upcycle these OPW into functional food products or incorporated in drug delivery devices [17] with potential bioactive properties for enhancing vascular health in animal models [18] or 3D printed biofabricated models [19].

Traditional extraction methods involve the use of organic solvents, such as methanol and ethanol, which are generally neither environmentally friendly nor sustainable. Furthermore, the use of organic solvents during the extraction process may increase costs of production, as the resultant organic wastes would require proper disposal [20,21,22]. In addition, compounds extracted using organic solvents would require further processing and purification to remove these solvents before they are safe to be consumed, increasing the overall complexity of the compound extraction process [20,21,22]. As a result, other green extraction methods that do not involved the use of organic solvents are developed for the extraction of bioactive compounds. These extraction methods include microwave-assisted extraction, deep eutectic solvent extraction, and pressurized hot water extraction (PHWE) [23,24,25]. While these methods are generally considered to be “greener” compared to traditional methods, they may also require different instrumentations and complex protocols that may affect their extraction efficiency, extraction yield, and energy consumption [25,26]. In the context of PHWE, water is used as the extraction solvent and is heated to temperatures between 100–374 °C under a constant pressure. This causes the dielectric constant associated with the polarity of water to reduce and enable the extraction of less polar compounds, resulting in improved extraction efficiency [25,26].

Therefore, the aim of the current study is to explore the use of PHWE, with water as an eco-friendlier alternative to organic solvents, in the extraction of bioactive compounds from OPW. The OPW extracts (OPWE) obtained using PHWE do not require an additional solvent removal step, reducing the overall complexity of the extraction method. Furthermore, as PHWE does require the presence of a simultaneously operating heating and cooling system, it may be a more energy-efficient compared to other methods [23,24,25,26]. Subsequently, the chemical profile and yield of bioactive compounds in the OPWE will be characterised along with their biological properties such as antioxidant activity, anti-inflammatory effects and metabolomic profile in endothelial cells. 

## 2. Materials and Methods

### 2.1. Sample Preparation and Chemicals

HPLC-grade water, formic acid, methanol, sand, 2,2′-Azinobis-3-ethylbenzothiazoline-6-sulfonic acid (ABTS), 1,1-diphenyl-2-picrylhydrazyl (DPPH), and standards used in HPLC were purchased from Sigma-Aldrich (Singapore). Fresh Australian navel OPW were donated by a local food vendor (UglyFood, Singapore). OPW were lyophilised and blended using a food blender into powder form and sieved to obtain powder of particle size <0.3 mm. The <0.3 mm powder was used for the respective extraction methods.

### 2.2. Extraction of Compounds Using PHWE and Methanolic Extraction

The PHWE system was assembled as previously described, consisting of a stainless-steel extraction column (250 mm × 10 mm i.d.), an isocratic LC10 series pump (Shimadzu, Japan), and a 5890 Series II temperature-controlled oven (Hewlett-Packard, Palo Alto, CA, USA) [27,28]. Stainless steel tubings of 1/16 in o.d. and 0.18 mm i.d. was used for all connections to ensure efficient heat transfer. Then, 0.75 g of sample were weighed and mixed with a small proportion of sand before loading into the extraction cell to maintain back pressure. The flow rate of the pump was set at a constant rate of 1.2 mL/min for 40–50 min. PHWE was carried out at 60, 80, 100, and 120 °C, respectively. Each extraction was carried out in triplicates, and 45 mL of extracts were collected during each repeat. For methanol extraction, 0.75 g of sample were weighed into 50 mL centrifuge tubes, and 25 mL of methanol were added into each tube. Tubes were placed in a sonicator water bath and subjected to sonication for 10 min at 40 °C in triplicates. All extracts were subsequently concentrated and lyophilised into dried powder using a freezer drier, which was stored for subsequent experiments.

### 2.3. LC/UV/MS Profiling of OPW

The chemical profiles of the respective OPWE were determined using LC/UV/MS as described previously. Briefly, for separation of the compounds, a longer C18 reverse phase HPLC column (Zorbax SB-C18 3.5 microns, 4.6 × 100 mm; Agilent, Santa Clara, CA, USA) was used for LC/UV, while a shorter column (Zorbax SB-C18 3.5 microns, 2.1 × 50 mm; Agilent, USA) was used for LCMS. The gradient elution consisted of a mobile phase involving 0.1% formic acid in (A) water and (B) acetonitrile, respectively. Detection of bioactive compounds based on the standards involved was detected by the UV detector at the absorbance value of 280 nm. For LC/UV, the calibration curve, coefficient of determination *r^2^*, and relative standard deviation (RSD) were performed. The calibration curve was obtained and would be based on for the prediction of analyte concentration based on the LC/UV’s response to the standards used. The *r^2^* values for all 5 standards used were at least 0.9990, indicating strong correlation between values obtained and actual values. RSD values (%) obtained for 6 consecutive injections of the respective analytes were ≤1.00%, indicating less variation between values and high repeatability of results. To normalise the peak areas to a constant sum, the measured peak intensity for each sample was normalised prior to obtaining an average reading for the three samples. For LC/UV, peaks were obtained based on the wavelengths at 254 nm. As for LC/MS, peak intensities obtained based on the molecular weights (*m*/*z*) and were normalised within each sample to the total peak intensity of the sample; normalisation was carried out to address concentration differences. The normalised peak area for each sample were further analysed using the principal component analysis (PCA) scores plot. The current approach was consistent with our earlier reports [27,28,29].

### 2.4. Antioxidant Activity Using DPPH Assay

The DPPH assay was used to determine the antioxidant activity of the OPW extracted using different temperatures of PHWE and methanol extraction as described previously [28,29]. Briefly, 100 µL of the respective OPWE (2–5000 mg/L) extracts or negative control (ddH_2_O) were transferred into a 96-well plate. Subsequently, 100 µL of 0.1 mM DPPH solution was pipetted into those wells and mixed well. For the positive control, 10 mM ascorbic acid was used in place of the OPWE. The plate was wrapped with aluminium foil and incubated at 25 °C for 30 min. After incubation, the Multiskan GO microplate reader (Thermo Scientific, Singapore) was used to record the absorbance at 517 nm. Readings were processed as below:

(1) $$\%\;\mathrm{maximum}\;\mathrm{inhibition}=\frac{\mathrm{An}-\mathrm{As}}{\mathrm{An}-\mathrm{Ap}}\times 100$$

where An refers to the negative control absorbance value, As refers to the sample absorbance measured, and Ap the absorbance measured from the ascorbic acid.

### 2.5. Antioxidant Capacity Using ABTS Assay

The vitamin C equivalent antioxidant capacity (CEAC) values of the OPWE were calculated and determined using ABTS assay as previously described [28]. Briefly, the standard curve of ascorbic acid (40 µM to 400 µM) were prepared that will produce between 5 to 50% scavenging of pre-formed radicals. Three replicates of OPWE (0.1–5 mg/mL) were measured by pipetting 25 µL of each sample to a 96-well microplate, followed by the addition of 200 µL ABTS^•+^ solution. The plate was incubated at 25 °C for 30 min in the Multiskan GO microplate reader (Thermo Scientific, Singapore), and absorbances were read at 734 nm. The CEAC values of the orange peel samples were calculated based on the equation obtained from the linear regression of the standard curve plotted.

### 2.6. Cell Culture and Treatment with OPWE

Human Dermal Microvascular Endothelium (HMEC-1) cells were obtained (American Type Culture Collection (ATCC), USA) and cultured in MCBD-131 media (20% foetal bovine serum (FBS), 5% L-glutamic acid (200 mM), 1% penicillin-streptomycin, and 0.001% recombinant human epidermal growth factor (EGF) (10 ng/mL)) using T75 flasks at 37 °C, 5% CO_2_. At 75–85% confluency, cells were seeded into cell-cultured plate under serum-starved condition (2% FBS) and incubated for 24 h. The HMEC-1 cells were treated with control (absence of TNF-α), OPWE alone (1 mg/mL), TNF-α (1 ng/mL), or TNF-α (1 ng/mL) co-incubated with OPWE (1 mg/mL) for 24 h. After treatment, the cells were harvested for cellular antioxidant, qPCR, or metabolomics experiments.

### 2.7. Measurement of Intracellular Oxidative Stress

After 24 h of treatment, the wells were rinsed with Hanks’ Balanced Salt Solution (HBSS) to removed cell debris and culture medium. The cells were incubated with 2′,7′-Dichlorofluorescin diacetate (DCFDA) (Cayman chemical, USA) (10 µM) at 37 °C, 5% CO_2_ for 60 min. Subsequently, the cells were rinsed 3X with HBSS to remove excess DCFDA and counter-stained with 10 µM Hoechst 33,342 for further 15 min at 37 °C, 5% CO_2_. After incubation, the cells were rinsed 3X with HBSS, and the plate was read with the Varioskan LUX multimode microplate reader (Thermo Scientific, Singapore) with the following excitation and emission wavelength for DCFDA (485 nm/520 nm) and Hoechst 33,342 (350 nm/461 nm). Readings from DCFDA levels were normalised to Hoechst 33,342 and expressed as fold change to control levels.

### 2.8. RNA Extraction and Quantitative Real-Time Polymerase Chain Reaction (qPCR)

After 24 h of treatment, the wells were washed with phosphate-buffered saline (PBS), RNA was extracted using the Aurum^TM^ Total RNA Mini Kit (Bio-Rad, Hercules, CA, USA), and the RNA quality and quantity were analysed as described earlier. Briefly, reverse transcription was carried out for the RNA samples in a single run with the T100^TM^ Thermal cycler (Bio-Rad, USA) to produce complementary DNA (cDNA) using the Bio-rad iScript cDNA Synthesis Kit. The final reaction volume was 20 µL, containing 0.5 µg of RNA. The comparative cycle threshold (2^−ΔΔCt^) method of qPCR was carried out according to earlier reports [30,31]. The CFX96 Real-time PCR system (Bio-rad, USA) was used to evaluate the relative gene expression of intracellular adhesion molecule 1 (*Icam-1*), vascular cell adhesion molecule 1 (*Vcam-1*), interleukin 1 Beta (*Il1β*), endothelin 1 (*Edn1*), prostaglandin-endoperoxide synthase 2 (*Ptgs2*), nitric oxide synthase 3 (*Nos3*), glutathione peroxidase 1 (*Gpx1*), superoxide dismutase 1 (*Sod1*), and nuclear factor erythroid 2-related factor 2 (*Nfe2l2*). qPCR was performed in 96-well plates with each well containing 10 µL volume reactions (10 µM primers and SYBR Green master mix (Bio-rad, USA)) prepared in triplicates. Glyceraldehyde-3-Phosphate Dehydrogenase (*Gapdh*) was used as the reference housekeeping gene. Negative template controls were prepared by substituting cDNA with water or by having the reverse transcriptase in the cDNA synthesis substituted with RT negative controls.

### 2.9. Lipid Extraction and Metabolomic Profiling of HMEC-1 Cells Using LC/MS

Lipids were extracted using chloroform:methanol method, and LCMS was performed as previously reported with minor modifications [32,33]. The extracted cell samples were vortex briefly and were centrifuged for 5 min at 10,000 rpm. The supernatant was transferred into a new microcentrifuge tube and dried in a vapour centrifuge for 30 min at 45 °C. Finally, 200 μL of methanol were added to the dried sample prior to analysis on LC/MS. A C18 reverse-phase HPLC column (Zorbax SB-C18- 3.5 microns, 2.1 × 100 mm) was used for LC/MS (Shimadzu LCMS-8050). A gradient elution was used involving a mobile phase (A) with 0.1% of formic acid in water and (B) 0.1% of formic acid in acetonitrile. For analysis of data, a targeted approach based on the measured peak intensity for the reading of each sample was used. For LC/MS, the peak intensities that correspond to the various molecular weight (*m*/*z*) are normalised within each sample to the total peak intensity of the sample. Normalisation was performed to address the differences in concentration. Additionally, normalized data for each sample were analysed using the PCA and orthogonal projection to latent structure discriminant analysis (OPLS-DA) scores plot for the selected peaks. The fatty acids, lipids, and other small molecules were identified based on our earlier work and comparison with reference standards [32,33].

### 2.10. Statistical Analysis

PCA and OPLS-DA plots were generated by the Soft Independent Modelling by Class Analogy (SIMCA) software. Concentration–response curves for DPPH inhibition were fitted using Graphpad Prism 6 software (GraphPad, San Diego, CA, USA) to generate a sigmoidal curve. Nonlinear regression was performed to calculate the sensitivity of DPPH inhibition (IC50) by the OPWE. Group mean values were analysed by one-way ANOVA with post hoc analysis using Tukey’s test. All data are presented as mean ± SEM. Statistical significance was considered when *p*-value was <0.05.

## 3. Results and Discussion

### 3.1. PHWE and Chemical Standardization of OPWE

**Figure 1:**
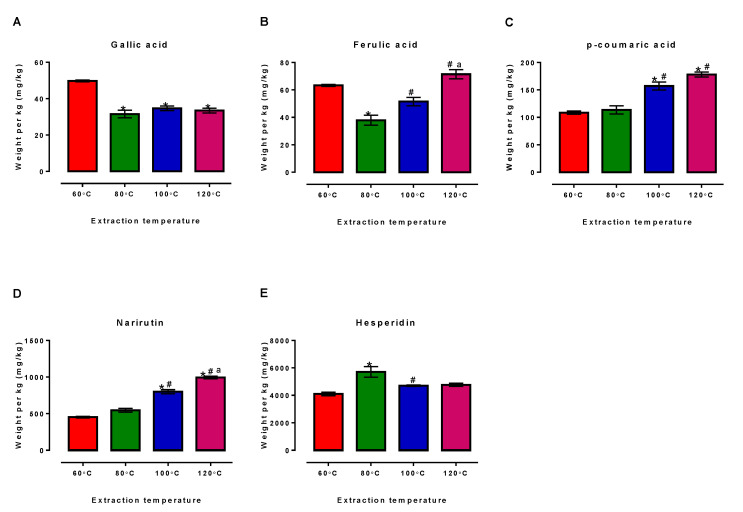
LCUV analysis of the amount of standard compounds, (**A**) gallic acid, (**B**) ferulic acid, (**C**) p-coumaric acid, (**D**) narirutin, and (**E**) hesperidin extracted using PHWE at different extraction temperatures. Each bar is represented by mean ± SEM, n = 3. *, Significantly different to 60 °C extraction temperature; #, significantly different to 80 °C extraction temperature; a, significantly different to 100 °C extraction temperature; *p* < 0.05, one-way ANOVA, Tukey’s post hoc test.

While there are several factors, such as applied temperature, particle size, flowrate, and solvent system, known to affect the extraction efficiency of PHWE, the applied temperature was observed to affect the yield of each compound [23,25]. As shown in Figure 1, different extraction temperatures resulted in different yields of several compounds detected. Specifically, for the current work, an applied temperature of 80 °C had resulted in the lowest gallic acid and ferulic acid yield (Figure 1A,B) although the yield of hesperidin was highest at this temperature (Figure 1E). While extraction at 60 °C reported the highest gallic acid and ferulic acid yield (Figure 1A,B), its p-coumaric acid and narirutin yield (Figure 1C,D) were reportedly the lowest amongst the other temperatures. Based on Figure 1B,D, the applied temperature at 120 °C was found to yield the highest amount of ferulic acid, p-coumaric acid, and narirutin, respectively.

The amount of hesperidin and narirutin determined were consistent with other studies at the same temperature, as these two flavones in orange peels were reported to be present in abundance in previous studies [2,34]. Most flavonoids, including hesperidin, are non-polar compounds and may be successfully extracted using water as extraction solvent. While water is a polar solvent, increasing the extraction temperature would decrease its dielectric constant and reduce the polarity of water, resulting in the extraction of non-polar compounds [23,25]. Indeed, previous studies have demonstrated that increasing in the extraction temperature during subcritical water extraction (SWE) had resulted in increased amounts of hesperidin and narirutin obtained [2,3,34], suggesting that higher PHWE temperatures have likely contributed to greater hesperidin yields (Figure 1E) when comparing extraction at 100 °C and 120 °C to that at 60 °C. Despite this, our study reported a much higher hesperidin yield with extraction at 80 °C compared to at 100 °C and 120 °C, which may have been due possible hydrolyzation of hesperidin to its aglycone isoform (hesperetin or hesperetin-7-*O*-glucoside) at the higher temperatures. This could also explain why the yield difference between extraction at 100 °C and at 120 °C were comparable. While higher temperature would reduce the polarity of water, resulting in lower solubility of compounds, this was not the case for narirutin and p-coumaric acid, as their yields had increased with temperature; intermolecular forces such as hydrogen, van der Waals, and dipole–dipole bonds may have been reduced, resulting in lower activation energy required for the desorption of extractable compounds. Increased diffusivity due to increased water temperature, together with the reduction of water’s surface tension, may have also improved the desorption of the two compounds from the matrix of the OPW sample [35,36]. Furthermore, as both p-coumaric acid and narirutin have a much higher water-solubility compared to hesperidin, the reduction of water polarity was proposed to have a lesser impact on their yields. To compare the extraction efficiency of PHWE against organic solvent extraction, the amount of target compounds extracted by PHWE were compared with methanol extraction (Table 1).

**Figure 2:**
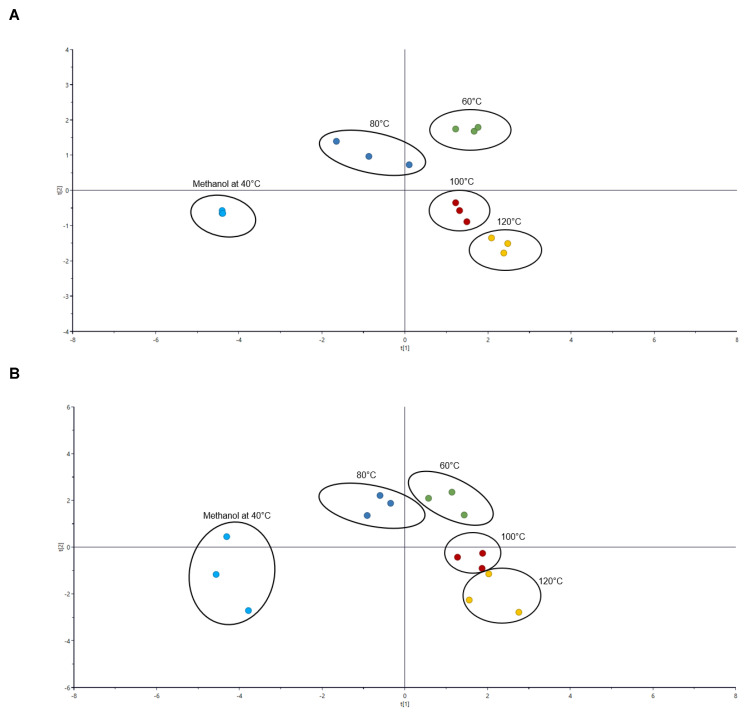
PCA score plot of (**A**) LC-UV profile at UV 254 nm and (**B**) LC-MS profile of orange peel extracts obtained from the different extraction conditions (PHWE 60 °C, PHWE 80 °C, PHWE 100 °C, PHWE 120 °C, Methanol 40 °C).

To obtain the chromatographic chemical fingerprint and to reduce bias due to factors such as possible differences between sample replicates and instrument variability and sample peaks obtained, chromatographic peaks from LC/UV and LC/MS were normalized such that the value obtained for each compound at the respective temperature was expressed as a proportion of the total peak area at that condition. With reference to Figure 2A, PHWE extraction at the respective temperatures and methanolic extraction had produced distinct profiles when analysed using LC-UV, based on known compounds listed in Table 1. Similarly, the profile obtained from LC-MS analysis (Figure 2B) was also relatively similar to that of LC-UV. Therefore, this further supports that extraction using PHWE would likely result in a different chemical fingerprint and concentration of target compounds as compared to using sonication with methanol (Table 1). The current approach and the data obtained were consistent with our earlier works, where a characteristic chemical fingerprint can be obtained from samples of different sources [28,29,37]. In addition, different PHWE temperatures would also result in a change in the chemical fingerprint, showing that the amount of various target compounds derived had varied (Figure 1).

### 3.2. Antioxidant Activity of OPWE

**Figure 3:**
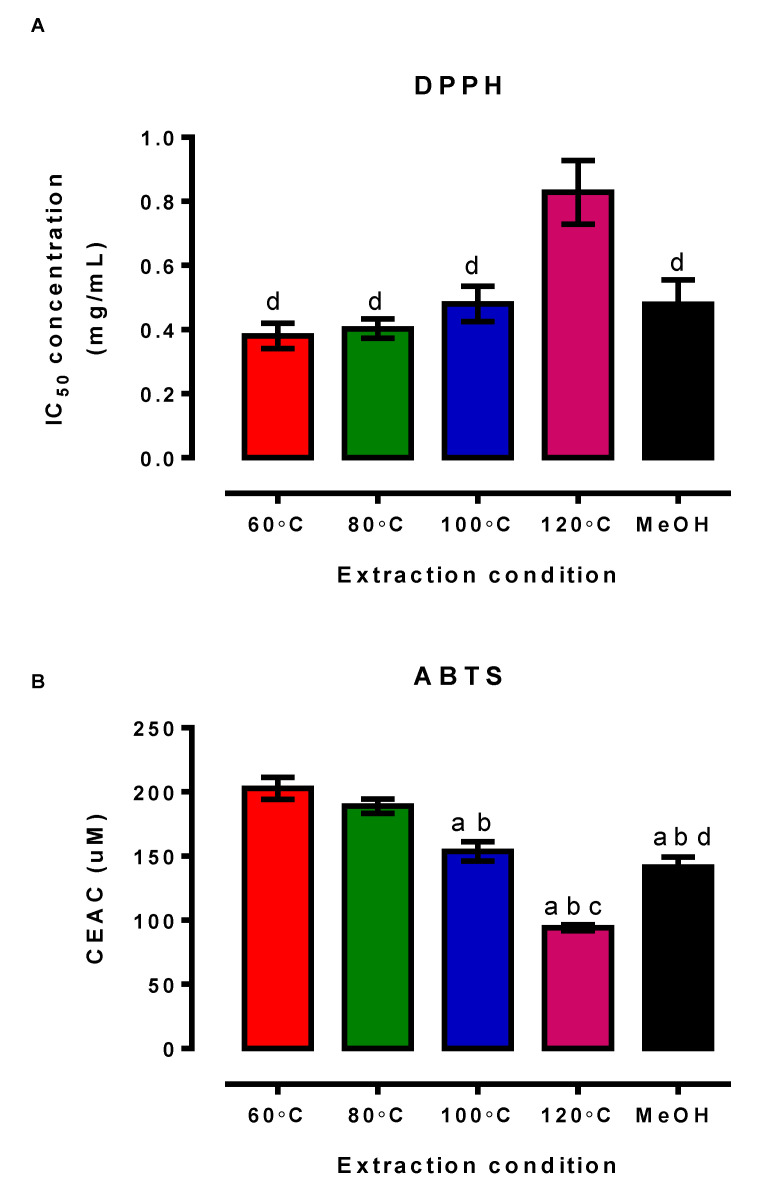
Antioxidant activity of OPWE obtained from different extraction conditions was evaluated two different assays, (**A**) DPPH and (**B**) ABTS. (**A**) Inhibitory concentration (IC50) value from concentration response curve of OPWE obtained from different extraction conditions. (**B**) CEAC of OPWE obtained from different extraction conditions. Each bar is represented by mean ± SEM, n = 5. ^a^, significantly different to 60 °C extraction temperature; ^b^, significantly different to 80 °C extraction temperature; ^c^, significantly different to 100 °C extraction temperature; ^d^, significantly different to 120 °C extraction temperature; *p* < 0.05, one-way ANOVA, Tukey’s post hoc test.

One well-reported biological activity of flavonoids is their potent antioxidant activity although it is unclear if the antioxidant activity of OPWE is affected by the different extraction method given the distinct chemical profile observed. Hence, we evaluated the antioxidant capacity of the OPWE from different PHWE temperatures and methanol extraction using two different antioxidant assays. Based on the DPPH and ATBS assays, OPWE using either PHWE or methanol extraction demonstrated antioxidant activity, which were consistent with earlier studies. However, in this study, we demonstrated that different PHWE temperatures exhibited different antioxidant efficacies. Specifically, the PHWE extracts at 60 °C and 80 °C reported comparable IC_50_ concentration, which were significantly lower compared to PHWE extracts at 100 °C and 120 °C, indicating that the antioxidant activity is more potent at lower extraction temperature. Both 100 °C and methanolic extracts reported relatively similar IC_50_ concentrations, which are also significantly less potent that PHWE extracts at 60 °C and 80 °C (Figure 3A). Similarly, results for the ABTS assay were consistent with DPPH assay (Figure 3B). Our results showed that CEAC for PHWE at the 60 °C and 80 °C was significantly higher compared to other PHWE temperature (100 °C and 120 °C) and methanol extraction (Figure 3B). One possible explanation for the observed differences in antioxidant capacity is likely to be different composition of flavonoids present in the OPWE. For example, in this study, PHWE at 60 °C extract had the highest gallic acid and ferulic acid concentration, which were further accompanied by highest antioxidant capacity. This is supported by a study that revealed that gallic acid had the highest antioxidant potency compared to other phenolic acids such as ferulic acid and p-coumaric acid [38]. Taken together, based on the yield of the respective compounds and antioxidant capacity, it remains inconclusive to attribute the antioxidant effect to certain compounds present in the OPWE, as there could be other compounds present in the OPWE that may have contributed to antioxidant activity that we did not detect in this study. Interestingly, an inverse relationship between temperature of PHWE and antioxidant activity was noted. Despite not being able to detect all the compounds present in the OPWE, we established the PHWE conditions (60 °C and 80 °C) that allowed the OPWEs to exhibit the best antioxidant capacity, which will be used for subsequent cellular experiments.

### 3.3. Effects of OPWE on TNFα-Induced Endothelial Dysfunction In Vitro

**Figure 4:**
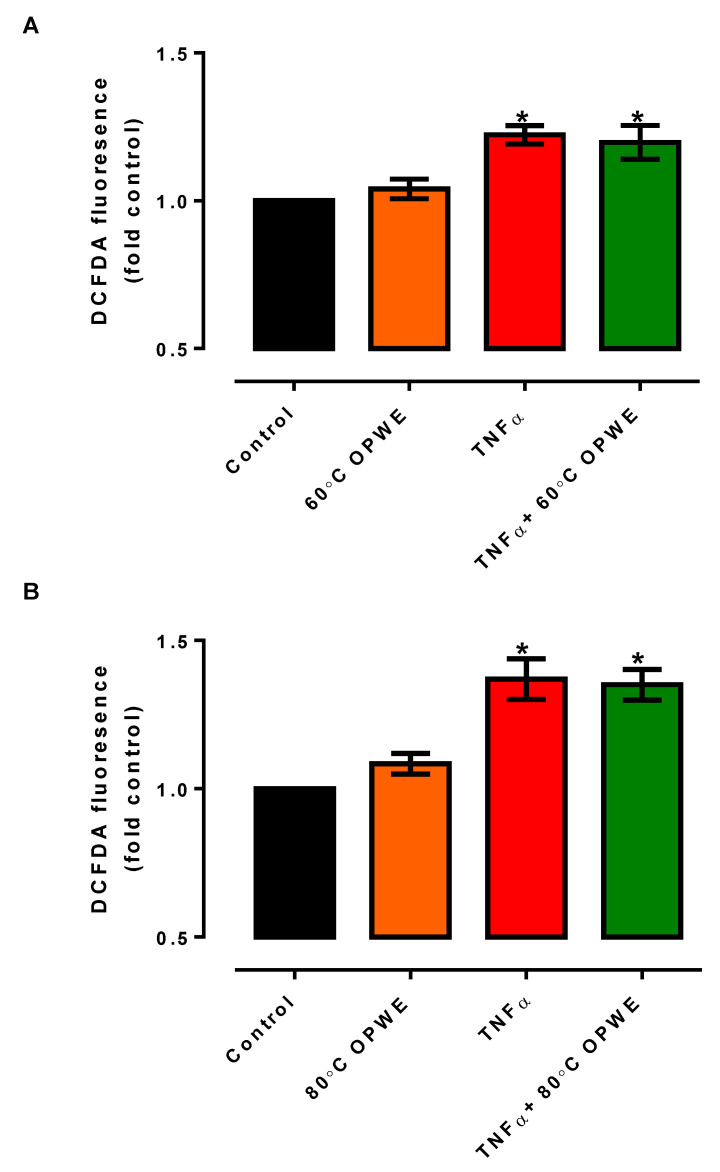
Intracellular levels of ROS in HMEC-1 cells in the absence (control) and in presence of either 1 mg/mL OPWE alone ((**A**): 60 °C PHWE, (**B**) 80 °C PHWE), 1 ng/mL TNFα alone, or the combination of TNFα + OPWE treatment for 24 h. Each bar is represented by mean ± SEM, n = 5. *, significantly different to control; *p* < 0.05, one-way ANOVA, Tukey’s post hoc test.

**Figure 5:**
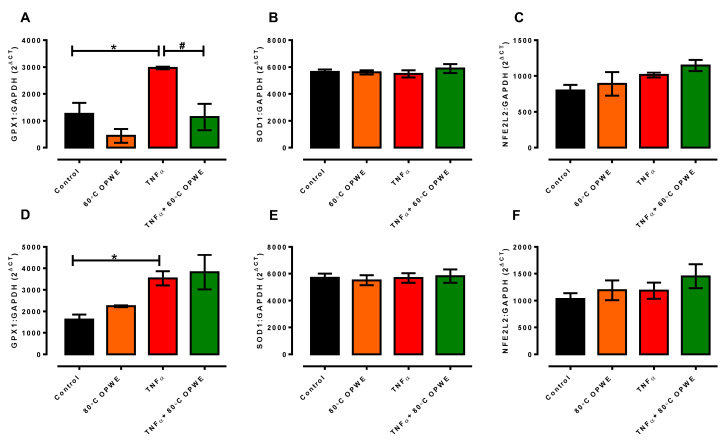
Quantitative analysis of (**A**,**D**) *Gpx1*, (**B**,**E**) *Sod1*, and (**C**,**F**) *Nfe2l2* mRNA expression in the absence (control) or in presence of either 1 mg/mL OPWE alone (A–C: 60 °C PHWE, D–F: 80 °C PHWE), 1 ng/mL TNFα alone, or the combination of TNFα + OPWE treatment for 24 h. Each bar is represented by mean ± SEM, n = 3–5. *, significantly different to control; #, significantly different to TNFα alone; *p* < 0.05, one-way ANOVA, Tukey’s post hoc test.

Oxidative stress and inflammation are thought to be important causes of endothelial dysfunction, which is recognised as an important contributing factor to vascular complications in diabetes, reproductive diseases, and hypertension [39,40,41,42,43]. One of the important inflammatory cytokines, TNF-α, is recognised to be released during inflammation, leading to the activation of various signalling pathways and resulting in endothelial dysfunction [44,45]. While OPWE is reported to possess antioxidant and anti-inflammatory properties, it is not known if OPWE treatment is able to reverse TNF-α-induced endothelial dysfunction in vitro. Consistent with earlier studies, treatment with TNF-α (1 ng/mL) for 24 h significantly increased oxidative stress in the endothelial cells, indicative of endothelial dysfunction (Figure 3). Interestingly, treatment with OPWE (1 mg/mL) from 60 °C (Figure 4A) or 80 °C (Figure 4B) did not have any significant effects on TNF-α-induced oxidative stress in the endothelial cells. As a result of enhanced oxidative stress, there was a compensatory upregulation of endogenous antioxidant enzyme, *Gpx1*, in the TNF-α-induced endothelial cells (Figure 5A). Co-treatment with 60 °C OPWE (Figure 5A) and not 80 °C OPWE (Figure 5D) significantly reduced the expression of Gpx1 to control levels. Neither TNF-α nor OPWE (60 °C and 80 °C) treatment had any effect on the gene expression of *Sod1* and *Nfe2l2* in the endothelial cells (Figure 5B–F). While the 60 °C and 80 °C OPWE were able to scavenge free radicals in cell-free assays (DPPH and ABTS), the antioxidant effects were not observed using intracellular reactive oxygen species assay.

**Figure 6:**
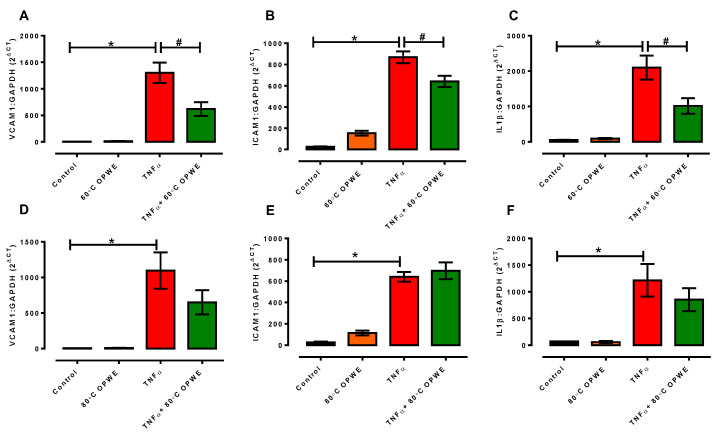
Quantitative analysis of (**A**,**D**) *vcam1*, (**B**,**E**) *icam1*, and (**C**,**F**) *Il1β* mRNA expression in the absence (control) or in presence of either 1 mg/mL OPWE alone (A–C: 60 °C PHWE, D–F: 80 °C PHWE), 1 ng/mL TNFα alone, or the combination of TNFα + OPWE treatment for 24 h. Each bar is represented by mean ± SEM, n = 3–5. *, significantly different to control; #, significantly different to TNFα alone; *p* < 0.05, one-way ANOVA, Tukey’s post hoc test.

To evaluate the effects of OPWE on TNF-α-induced endothelial dysfunction, we measured the gene expression of adhesion molecules, *vcam-1* and *icam-1*, and inflammatory cytokine, *Il1β*, in the endothelial cells. In this study, TNF-α caused significant upregulation of the gene expression of *vcam-1*, *icam-1*, and *Il1β* in the endothelial cells (Figure 6). Neither treatment with 60 °C nor 80 °C OPWE, respectively, had any effect on the expression of *vcam-1*, *icam-1*, and *Il1β*. Interestingly, only the 60 °C OPWE (Figure 6A–C) co-treatment had significantly reduced the TNF-α-induced upregulation of the gene expression of *vcam-1*, *icam-1*, and *Il1β* in the endothelial cells. This suggests that only 60 °C OPWE but not 80 °C OPWE exhibited anti-inflammatory effects in the endothelial cells.

**Figure 7:**
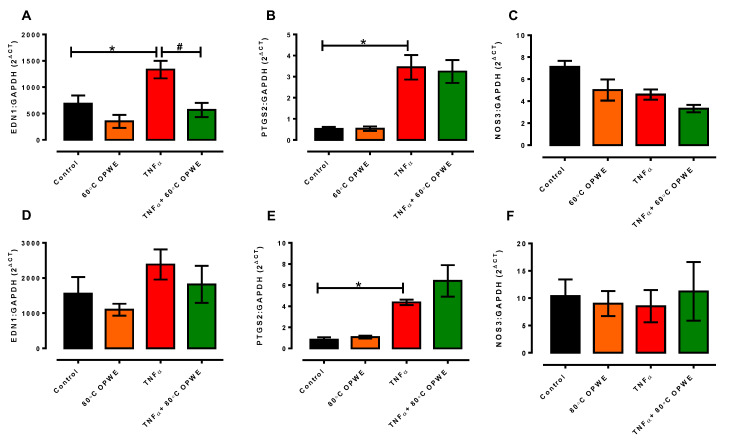
Quantitative analysis of (**A**,**D**) *Edn1*, (**B**,**E**) *Ptgs2*, and (**C**,**F**) *Nos3* mRNA expression in the absence (control) or in presence of either 1 mg/mL OPWE alone ((**A**–**C**): 60 °C PHWE, (**D**–**F**): 80 °C PHWE), 1 ng/mL TNFα alone, or the combination of TNFα + OPWE treatment for 24 h. Each bar is represented by mean ± SEM, n = 3–5. *, significantly different to control; #, significantly different to TNFα alone; *p* < 0.05, one-way ANOVA, Tukey’s post hoc test.

Endothelial cells are known to regulate vascular tone through the secretion of several vasoactive factors such as endothelin-1, prostanoids, and nitric oxide [46]. Endothelial dysfunction results from the overproduction of vasoconstrictor factors and/or diminished production of vasodilator factors [47,48]. In this study, TNF-α treatment significantly increased the gene expression of *Edn1* and *Ptgs2* but had no effect on *Nos3* expression, suggesting the upregulation of the vasoconstrictor *Edn1* (Figure 7). Co-treatment of 60 °C OPWE with TNF-α suppressed the gene expression of *Edn1* (Figure 7A) but not the expression of *Ptgs2* or *Nos3* (Figure 6B,C). However, neither treatment with 80 °C OPWE alone nor co-incubated with TNF-α had any effect on the gene expression of *Edn1*, *Ptgs2*, and *Nos3* in the endothelial cells (Figure 7D–F). Overall, our study demonstrated that PHWE at 60 °C OPWE exhibited anti-inflammatory activity and reduced TNF-α-induced endothelial dysfunction in vitro.

**Figure 8:**
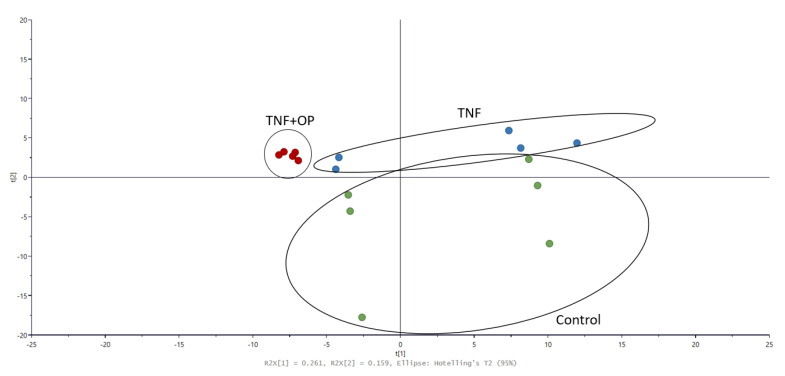
PCA score plot of the metabolic profile of HMEC-1 cells after 24 h treatment of control: 60 °C OPWE (1 mg/mL) or co-treated with TNF-α (1 ng/mL) and 60 °C OPWE (1 mg/mL). Some of the key metabolites determined are shown in Table 2 (n = 5–6).

The presence of oxidative stress and inflammation were reported to cause changes in the lipid profile, contributing to endothelial dysfunction. Therefore, to further characterize the potential vascular protective effects of 60 °C OPWE in the context of TNF-α-induced endothelial dysfunction, metabolite profiling of several lipid markers was carried out (Figure 8, Table 2). The PCA score plot showed distinctive metabolite profile between the control, TNF-α treatment alone, and TNF-α co-treated with 60 °C OPWE (Figure 8). However, a distinctive metabolic profile was observed between the control and TNF-α treatment, indicating a marked alteration of metabolites in the endothelial cells (Figure 8). Interestingly, despite exhibiting anti-inflammatory activity, co-treatment with 60 °C OPWE and TNF-α shifted the metabolic profile further away from the control group, suggesting the 60 °C OPWE may have caused further changes to metabolic profile under TNF-α-stimulated conditions. While TNF-α treatment caused limited changes to the metabolites, choline, and acetylcarnitine, co-treatment with 60 °C OPWE significantly reduced the levels of these metabolites, suggesting that the effects of 60 °C OPWE on these metabolites were independent of TNF-α to regulate energy metabolism. Interestingly, the levels of the bile acid, deoxycholic acid was also significantly reduced by TNF-α treatment and was further suppressed when co-treated with 60 °C OPWE (Table 2). To date, the role of bile acids on endothelial cells remains controversial; while some studies have reported the pro-inflammatory effects of bile acids [49,50], others have demonstrated their vasoprotective effects [51,52]. Specifically, an earlier study reported that bile acids had enhanced adhesion molecule expression in endothelial cells [50] through oxidative stress and activation of NF-kappaB and p38 signalling pathway [49]. This suggested that elevated levels of bile acids may cause endothelium dysfunction and contribute to the initiation of early events of vascular inflammation [49]. In this study, TNF-α treatment also upregulated the expression of adhesion molecule in the endothelial cells, which may trigger a compensatory suppression bile acid level. Furthermore, in response to OPWE treatment, the levels of bile acids were further reduced, further accompanied by reduction in the expression of adhesion molecules. Taken together, OPWE treatment may reduce TNFα-induced inflammation and reverse endothelial dysfunction in vitro, which may be in part underpinned by the reduction in bile acid production.

## 4. Conclusions

OPW consists of a rich source of essential constituents that can be transformed into highly value-added bioproducts. Indeed, a plethora of studies have evaluated the potential to extract many different valuable bioactive compounds from OPW, including essential oils, pectin, and carotenoids. Specifically, other bioconversion technologies or additive manufacturing techniques have also been explored to convert OPW into biochemicals, biopolysaccharides, and bioenergy [3,15,24,34]. More recently, due to advancement of additive manufacturing techniques, other food products or food wastes including OPW may be explored to create 3D printed food [53,54,55,56,57]. While many studies employed different extraction methods to valorise polyphenols and other flavonoids from OPW due to their beneficial biological effects, such as antioxidant and anti-inflammatory effects, more sustainable methods of extraction, such as PHWE, may be considered. In this study, we demonstrated that OPWE using PHWE at 60 °C yielded distinct polyphenolic compounds comparable to organic solvent extraction but with superior antioxidant activity. Furthermore, treatment of cells with 60 °C OPWE reversed TNF-α-induced endothelial dysfunction in vitro primarily through anti-inflammatory action, suggesting the potential to upcycle OPW as a natural product to promote vascular health.

## Figures and Tables

**Table 1:** Amount of various compounds obtained by PHWE at 60 °C and methanolic extraction.

Compound Detection Wavelength (nm)		PHWE Extraction at 60 °C ^a^				Methanolic Extraction ^b^			
		Day 1		Day 2		Day 1		Day 2	
		Mean Weight ± SEM (mg/kg)	RSD%	Mean Weight ± SEM (mg/kg)	RSD%	Mean Weight ± SEM (mg/kg)	RSD%	Mean Weight ± SEM (mg/kg)	RSD%
Gallic acid	213	214.56 ± 26.15	27.25	133.40 ± 7.80	13.08	230.03 ± 6.02	4.53	84.89 ± 0.95	1.94
p-coumaric acid	223	112.82 ± 3.68	7.28	125.10 ± 2.40	4.29	109.75 ± 3.20	5.05	87.21 ± 3.25	6.45
Ferulic acid	323	98.33 ± 4.51	10.25	94.50 ± 6.55	15.49	394.26 ± 9.88	4.34	419.15 ± 5.43	2.24
Narirutin	284	900.22 ± 34.28	8.52	924.10 ± 17.44	4.22	140.16 ± 2.08	2.57	151.40 ± 6.26	7.16
Hesperidin	284	35,702.84 ± 2865.00	17.95	50,310.00 ± 1399.00	6.22	278,947.39 ± 2302.00	1.43	418,680.94 ± 7729.00	3.20

Derivation of data in this table was based on the above detection wavelength of the individual compounds. ^a^ Derived mean weight ± SEM and RSD for each compound were based on n = 5. ^b^ Derived mean weight ± SEM and RSD for each compound were based on n = 3.

**Table 2:** Analysis of metabolites of HMEC-1 cells after 24 h treatment of control: 60 °C OPWE (1 mg/mL) or co-treated with TNF-α (1 ng/mL) and 60 °C OPWE (1 mg/mL).

Retention Time/min	*m*/*z*	Identified Compounds	Normalized Peak Intensity (%)		
			Control	TNF-α (1 ng/mL)	TNF-α (1 ng/mL) + 60 °C OPWE (1mg/mL).
0.60	524.5	Unknown	0.000740 ± 0.000127	0.000824 ± 0.000142	0.000609 ± 0.0000315 *^b^*
0.78	104.2	Choline	0.0363 ± 0.0087	0.0325 ± 0.0059	0.0244 ± 0.00328 *^b^*
0.80	118.2	Betaine	0.00235 ± 0.000172	0.00236 ± 0.00015	0.00214 ± 0.00031
0.83	204.2	Acetylcarnitine	0.00126 ± 0.00064	0.00155 ± 0.00048	0.0009888 ± 0.000036 *^b^*
0.85	258.3	Glycerophosphocholine	0.00354 ± 0.0012	0.00265 ± 0.00082	0.00187 ± 0.00012
3.00	520.4	LPC C18:2	0.352 ± 0.0226	0.348 ± 0.0277	0.320 ± 0.0124
6.70	438	LTE4	0.0000165 ± 0.0000405	-	-
7.71	391	Deoxycholic acid	0.00778 ± 0.00378 *^b^*	0.00305 ± 0.00194	0.00038 ± 0.00012 *^b^*
8.40	319	19(s)-HETE/1 TR HETE	0.000210 ± 0.000173	0.000184 ± 0.000169	0.0000246 ± 0.0000549
8.50	496.4	LPC C16:0	0.00397 ± 0.0022 *^b^*	0.0106 ± 0.0025	0.0353 ± 0.0073 *^b^*
9.60	464	Unknown	0.00206 ± 0.000313	0.00201 ± 0.000303	0.00160 ± 0.000417 *^b^*
11.00	303	Arachidonic acid	0.000249 ± 0.000137	0.000152 ± 0.000105	0.000220 ±0.000130
11.30	255	Palmitic acid	0.000223 ± 0.000234	0.000505 ± 0.000355	0.0002267 ± 0.000159
11.60	303	Unknown	0.00149 ± 0.000249	0.00165 ± 0.000320	0.00122 ± 0.0000824 *^b^*
11.60	568	Unknown	0.00104 ± 0.000892	-	-

Values are represented as mean ± SD, n = 5–6. *^b^*, significant different from TNF-α treatment alone (*p* < 0.05).

## Data Availability

Not applicable.
